# Accelerometer-derived physical activity patterns and incident type 2 diabetes: a prospective cohort study

**DOI:** 10.1186/s12966-025-01734-7

**Published:** 2025-03-31

**Authors:** Dan-Qing Liao, Hao-Jie Chen, Hong-Min Li, Jian Gao, Xu-Lian Tang, Li-Ying Du, Shu-Min Lai, Wen-Fang Zhong, Hong-Xuan Huang, Zhi-Yuan Xiong, Pei-Liang Chen, Ling Kuang, Bing-Yun Zhang, Jin Yang, Qing-Mei Huang, Dan Liu, Pei-Dong Zhang, Chen Mao, Zhi-Hao Li

**Affiliations:** 1 Department of Epidemiology, School of Public Health, Southern Medical University, No.1023-1063, South Shatai Road, Guangzhou, 510515 Guangdong China; 2 Department of Neurosurgery, Institute of Brain Diseases, Nanfang Hospital, Southern Medical University, Guangzhou, Guangdong China

**Keywords:** Physical activity pattern, Weekend warrior, Type 2 diabetes, Accelerometer, Prospective study

## Abstract

**Background:**

Emerging evidence suggests a significant relationship between the duration of physical activity (PA) and the incidence of type 2 diabetes (T2D). However, the association between the “weekend warrior” (WW) pattern—characterized by concentrated moderate-to-vigorous PA (MVPA) over one to two days—and T2D remains unclear.

**Methods:**

This prospective cohort study aims to utilize device-measured PA data to investigate the associations between PA patterns and T2D. Individuals were divided into three MVPA patterns on the basis of WHO guidelines: inactive (< 150 min), active WW (≥ 150 min with ≥ 50% of total MVPA achieved in one to two days), and active regular (≥ 150 min but not active WW). These patterns were also evaluated using sample percentile thresholds. The relationships between PA patterns and the risk of T2D were analysed employing Cox proportional hazards models.

**Results:**

A total of 1972 participants developed T2D over a 7.9–year median follow-up period. In the fully adjusted model, both active patterns demonstrated comparable reductions in the risk of developing T2D (active WW: hazard ratio [HR] 0.64, 95% confidence interval [CI] 0.58–0.71; active regular: 0.56, 0.49–0.64). Moreover, the risk of T2D exhibited a progressive decline as the duration of MVPA increased across both active patterns.

**Conclusions:**

Engaging in MVPA for one or two days per week provides comparable protective benefits against the incidence of T2D as more evenly distributed PA. Additionally, exceeding the current guidelines may confer even greater advantages.

**Supplementary Information:**

The online version contains supplementary material available at 10.1186/s12966-025-01734-7.

## Background

Type 2 diabetes (T2D) represents a significant contributor to mortality and disability globally [1]. Currently, over 463 million adults are living with T2D, and projections indicate that this number may rise to 800 million by 2045 [2, 3]. This increase imposes significant economic and health burdens on individuals and society [4, 5], leading to the identification of risk factors, particularly those that are modifiable, and the implementation of effective preventive strategies as public health priorities. Previous studies suggest that engaging in physical activity (PA) is associated with a lower risk of T2D [6, 7].

Guidelines from the World Health Organization (WHO) and the American Heart Association advise at least 150 min of moderate-to-vigorous PA (MVPA) weekly, with more than 300 min advised for additional health benefits [8, 9]. However, these guidelines do not delineate an optimal distribution pattern for MVPA throughout the week. There is a noticeable research gap regarding the frequency aspect of MVPA. Individuals who concentrate their PA into one or two days are referred to as “weekend warriors” (WWs) [10]. Many adults worldwide adopt this exercise pattern due to time constraints [11]. Therefore, investigating the potential association between the WW pattern and T2D risk is highly significant for public health.

Studies exploring the relationships between various PA patterns and health outcomes are limited [12–17]. One study indicated that regularly active individuals and those engaging in the WW pattern had a lower risk of metabolic syndrome compared to inactive counterparts [12]. The results from three cohort studies demonstrated similar mortality benefits for individuals adhering to the WW pattern and those participating in regular activity [18–20]. However, these studies relied on self-reported PA data, which are prone to recall bias and measurement inaccuracies, potentially skewing the true relationship between the WW pattern and health outcomes [21]. Few investigations have examined the WW pattern using accelerometer data, with existing studies focused primarily on brain health [14, 15, 17], mortality [13], and cardiovascular illnesses [16]. Notably, no study has explored the relationship between objectively measured PA data and T2D risk.

Therefore, we utilized a subcohort from the UK Biobank comprising approximately 90,000 individuals invited to wear accelerometers to collect PA data. This study aimed to examine the associations between PA patterns and T2D risk, considering both PA duration and frequency, while also investigating factors that may influence these relationships.

## Methods

### Study design and participants

**Fig. 1:**
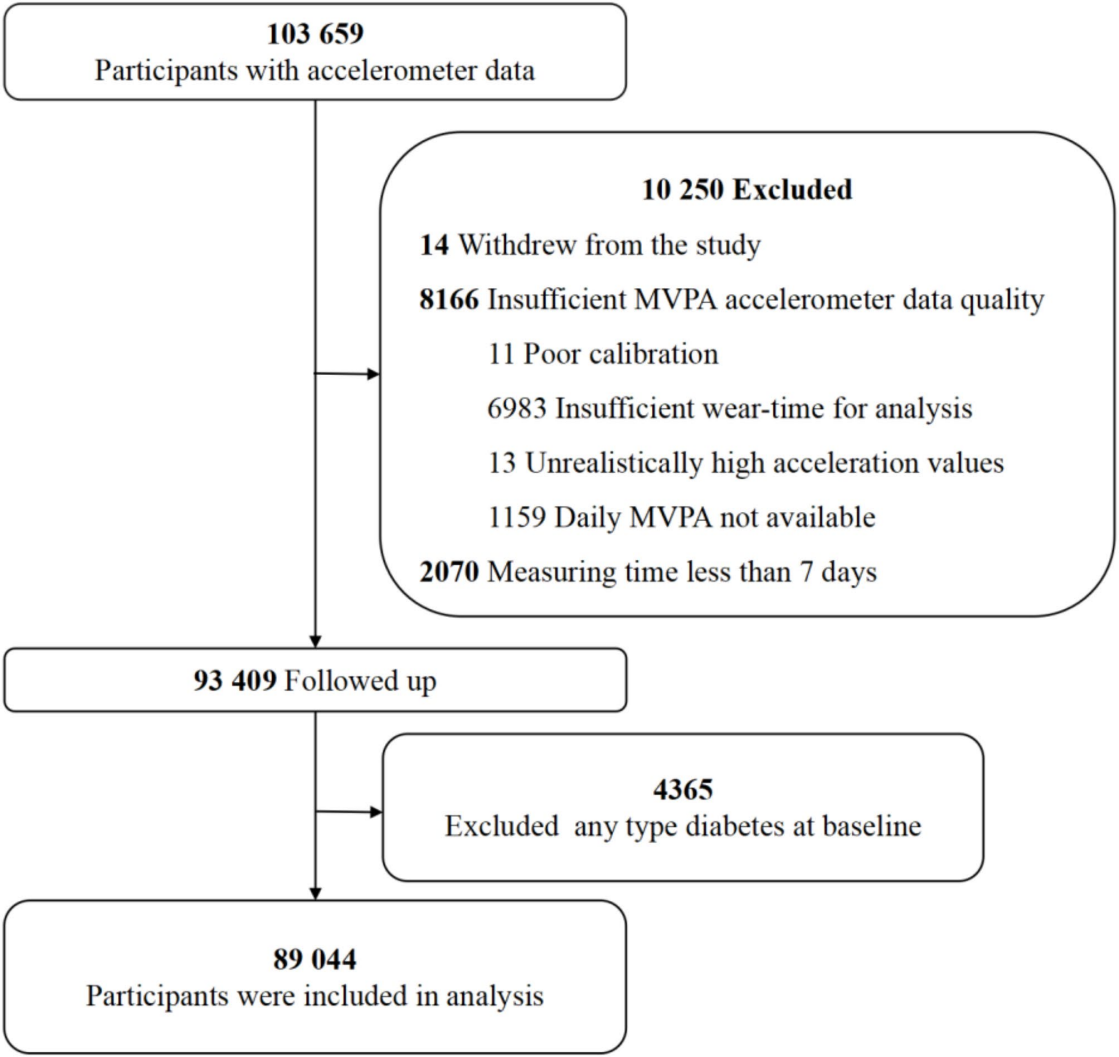
Flowchart of participant enrolment

The data employed in the present study were obtained from the UK Biobank. Prior research has described the UK Biobank study design and population [22, 23]. In brief, from 2006 to 2010, more than 500,000 individuals aged 40 to 69 years were recruited from the general population across the UK. The participants’ information including demographic characteristics, lifestyle, health data and biological samples was collected. The UK Biobank data is openly accessible to approved applicants (www.ukbiobank.ac.uk). For this analysis, individuals who withdrew from the study (*n* = 14), had poor-quality accelerometer data (*n* = 8166), or wore accelerometers for fewer than seven days (*n* = 2070) were excluded. Moreover, 4365 participants with any type of diabetes at baseline were further excluded, leaving 89,044 participants for the final analysis **(**Fig. 1**)**.

### Definition of PA patterns

The accelerometer substudy invited participants to wear Axivity AX triaxial accelerometers [24] on their dominant hands for seven days. These devices record data at 100 Hz with a dynamic range of ± 8 Hz. The acceleration signals were calibrated to gravity and recorded as 5–second epochs. Nonwear time was identified as stationary episodes longer than 60 min across all three axes with a standard deviation (SD) under 13.0 mg. Nonwear epochs were imputed via mean vector magnitude and intensity data from similar times on other days. For more information on the data collection and processing methods, refer to a previous study [24]. MVPA were determined using a classification method based on machine learning, developed to analyse a wide range of activities and validated as part of the UK Biobank study [25].

To address ambiguities in appropriate MVPA levels measured by wrist-worn accelerometers, we used the WHO guidelines of MVPA ≥ 150 min/week as a primary criterion and analysed additional thresholds within the sample to ensure the reliability of the results. The subjects were divided into three groups on the basis of WHO guidelines in this study: inactive (MVPA < 150 min weekly), active “weekend warrior” (WW) (MVPA ≥ 150 min weekly, with ≥ 50% of total MVPA achieved in one to two days), and active regular (MVPA ≥ 150 min weekly but not active WW). The same patterns were assessed via sample percentile thresholds: the 25th percentile (MVPA ≥ 115.2 min/week), the median (MVPA ≥ 230.4 min/week), and the 75th percentile (MVPA ≥ 403.2 min/week).

### Ascertainment of outcome

Diabetes prevalence was assessed using self-reported data and baseline HbA1c concentrations ≥ 6.5% to identify undiagnosed diabetes, as well as self-reported diabetes medication use at recruitment. Additionally, data were extracted from hospital episode statistics (HES) and Scottish Morbidity Records (SMR), which included events coded E10-E14 according to the International Classification of Diseases, 10th Revision (ICD-10), prior to accelerometry [26]. Incident T2D was ascertained through linkage with the HES (England and Wales) and SMR (Scotland) registers [22], and follow-up data was available until 28 October 2022. Therefore, the follow-up time was calculated from the completion of an accelerometer assessment to admission, death, or the censoring date, whichever occurred first. The following ICD codes were used to determine the incidence of T2D: code 250 in the ICD-9 and code E11 in the ICD-10.

### Assessment of covariates

Potential covariates were selected on the basis of previous research [16, 27] and included age (continuous, years), sex (female or male), ethnicity (white or other), education status (degree or no degree), Townsend Deprivation Index (TDI, continuous), household income (<£18,000, £18,000-£30,999, £31,000-£51,999, £52,000-£100,000, or >£100,000), employment status (employed or unemployed/retired), smoking status (current, previous, or never), drinking status (current, previous, or never), body mass index (BMI, underweight, normal weight, overweight, or obese), healthy dietary pattern status (yes or no) [28], sedentary time (categorized into low, moderate, or high based on tertile), sleep duration (< 7, 7–8, or > 8), cancer status (yes or no), CVD status (yes or no), hypertension status (yes or no), and parental history of diabetes (yes or no). The variable definitions are given in Table S1 and are available on the UK Biobank website (www.ukbiobank.ac.uk). To address the issue of missing covariate data, multiple imputations with chained equations were used under the assumption of random missing data [29]; see Table S2 for details.

### Statistical analysis

Baseline characteristics corresponding to participants’ PA patterns are presented as means (SDs) for continuous variables and as numbers (percentages) for categorical variables. To compare baseline characteristics across different PA patterns, we used the analysis of variance for continuous variables and the χ^2^ test for categorical variables. Multivariate Cox proportional hazards models were constructed to examine the associations between PA patterns and T2D risk, and hazard ratios (HRs) and 95% confidence intervals (CIs) were calculated to quantify these associations. We also investigated these associations stratified by the duration of MVPA per week. On the basis of guideline recommendations for PA, we categorized PA into four stratums: <150 min per week (inactive), from 150 to 300 min per week, from 300 to 600 min per week, and more than 600 min per week. Two models were used for analysis: Model 1 was adjusted for age and sex, and the fully adjusted model (Model 2) was further adjusted for ethnicity, education status, household income, TDI, employment status, smoking status, drinking status, BMI, healthy dietary pattern status, sedentary time, sleep duration, cancer status, CVD status, hypertension status, and parental history of diabetes. The proportional hazard assumption was evaluated by Schoenfeld residuals [30], and no violations of this assumption were found. Additionally, we compared the T2D risk between the WW group and the regularly active group with the same amount of MVPA per week.

We performed stratified analyses to explore potential variations in associations across these factors: sex (male or female), age (< 65 years or ≥ 65 years), obesity status (yes or no), education status (degree or no degree), current smoking status (yes or no), current drinking status (yes or no), CVD status (yes or no), hypertension status (yes or no), and sedentary time (high, above median; or low, below median). Interaction effects were assessed via likelihood ratio tests [31]. Moreover, we conducted several sensitivity analyses to ensure the robustness of the findings. First, we excluded patients with T2D occurring within the first two years of follow-up. Second, analyses were repeated using the unimputed dataset. Third, we varied the thresholds for defining the active WW group. Specifically, one WW definition considers WWs as meeting the weekly MVPA recommendation of 150 min, with at least 75% of the activity occurring on one to two days [17]. Another WW definition involved the achievement of 150 min of MVPA weekly, where at least 50% of this activity was achieved over one to two consecutive days [32]. Finally, we defined a WW group as those who achieved the 150-minute MVPA weekly goal, with at least 50% of their activity occurring on one or two weekend days. To assess the potential impact of unmeasured confounders on the observed associations, we calculated E values. E values quantify the magnitude of unmeasured confounding needed to explain away the observed relationship between PA patterns and incident T2D. A higher E value suggests that a stronger unmeasured confounder would be required to nullify the observed effect, thereby providing an estimate of the robustness of the observed associations. We also showed examples of the factors (obesity, smoking, and education) that E-values are designed to calculate limits for [33]. 

All the statistical analyses were conducted via R software (version 4.3.1). A two-sided *P* value < 0.05 was considered to indicate statistical significance. To account for multiple testing in interaction analyses, we applied Bonferroni correction, resulting in a significance threshold of *P* < 0.0056 (0.05/9) [34, 35].

## Results

### Characteristics of participants

**Fig. 2:**
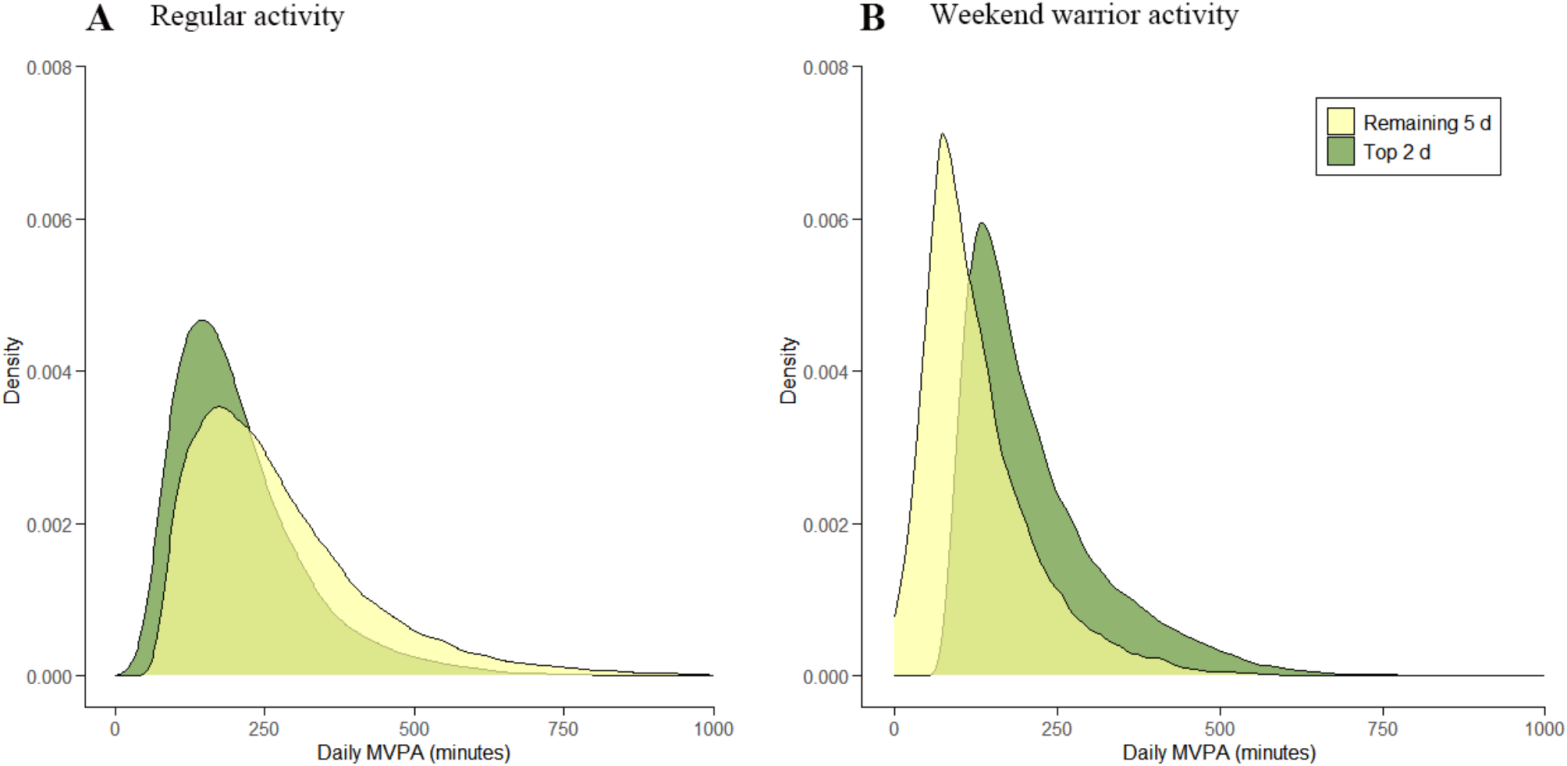
The distribution of daily MVPA on the 2 most active days of the week vs. the remaining 5 days using a guideline-based activity threshold of 150 min or more MVPA per week. **A** Regular activity and **B** Weekend warrior activity. “Top 2 d” means the sum of the two days with the most MVPA time in a week; “Remaining 5 d” means the total MVPA time for the remaining 5 days

Table 1 presents the baseline characteristics of the study participants stratified by PA patterns. Among the 89,044 participants included (mean [SD] age 62.3[7.9] years; 57.2% female; 97.1% white), 38,481 participants (43.2%) were classified as active WW, 21,300 participants (23.9%) as active regular, and 29,263 participants (32.9%) as inactive. Compared to the inactive group, WWs exhibited a lower percentage of females, higher income levels, fewer recent smokers, and longer weekly durations of MVPA. A density map comparing the daily MVPA distributions of the active regular and WW groups illustrated that the MVPA of the active regular group was more evenly distributed, while the active WW group showed greater MVPA on the top two days compared to the remaining five days **(**Fig. 2**).**

**Table 1:** Baseline characteristics of the study participants stratified by PA pattern

Characteristics	Active WW (*n* = 38481)	Active regular (*n* = 21300)	Inactive (*n* = 29263)	Overall (*n* = 89044)	*P* value
Age (years), mean (SD)	62.19 (7.75)	61.10 (7.91)	63.16 (7.86)	62.25 (7.86)	< 0.001
Female	19,899 (51.7)	11,006 (51.7)	20,007 (68.4)	50,912 (57.2)	< 0.001
Ethnicity					
White	37,539 (97.6)	20,585 (96.6)	28,313 (96.8)	86,437 (97.1)	< 0.001
Others	942 (2.4)	715 (3.4)	950 (3.2)	2607 (2.9)	
Education					
Degree	18,122 (47.1)	10,673 (50.1)	10,266 (35.1)	39,061 (43.9)	< 0.001
No degree	20,359 (52.9)	10,627 (49.9)	18,997 (64.9)	49,983 (56.1)	
Townsend Deprivation Index, mean (SD)	-1.94 (2.70)	-1.33 (2.99)	-1.83 (2.75)	-1.76 (2.80)	< 0.001
Household income (£) *					
Less than 18,000	4584 (11.9)	2617 (12.3)	5023 (17.2)	12,224 (13.7)	< 0.001
18,000 to 30,999	9286 (24.1)	5063 (23.8)	8228 (28.1)	22,577 (25.4)	
31,000 to 51,999	11,662 (30.3)	6251 (29.3)	8978 (30.7)	26,891 (30.2)	
52,000 to 100,000	10,028 (26.1)	5610 (26.3)	5807 (19.8)	21,445 (24.1)	
Greater than 100,000	2921 (7.6)	1759 (8.3)	1227 (4.2)	5907 (6.6)	
Employment status					
Employed	24,383 (63.4)	14,421 (67.7)	16,793 (57.4)	55,597 (62.4)	< 0.001
Unemployed/Retired	14,098 (36.6)	6879 (32.3)	12,470 (42.6)	33,447 (37.6)	
Smoking status					
Never	22,773 (59.2)	12,394 (58.2)	16,280 (55.6)	51,447 (57.8)	< 0.001
Previous	13,508 (35.1)	7586 (35.6)	10,439 (35.7)	31,533 (35.4)	
Current	2200 (5.7)	1320 (6.2)	2544 (8.7)	6064 (6.8)	
Drinking status					
Never	834 (2.2)	545 (2.6)	1141 (3.9)	2520 (2.8)	< 0.001
Previous	851 (2.2)	557 (2.6)	933 (3.2)	2341 (2.6)	
Current	36,796 (95.6)	20,198 (94.8)	27,189 (92.9)	84,183 (94.5)	
Healthy dietary pattern status	22,901 (59.5)	12,868 (60.4)	17,132 (58.5)	52,901 (59.4)	< 0.001
Body mass index, mean (SD), kg/m^2^	26.06 (3.89)	25.67 (3.91)	27.72 (4.96)	26.51 (4.36)	< 0.001
Body mass index category, kg/m^2^					
Underweight	215 (0.6)	187 (0.9)	121 (0.4)	523 (0.6)	< 0.001
Normal weight	16,367 (42.5)	10,049 (47.2)	9216 (31.5)	35,632 (40.0)	
Overweight	16,487 (42.8)	8371 (39.3)	12,029 (41.1)	36,887 (41.4)	
Obesity	5412 (14.1)	2693 (12.6)	7897 (27.0)	16,002 (18.0)	
Cancer	2622 (6.8)	1379 (6.5)	2476 (8.5)	6477 (7.3)	< 0.001
Cardiovascular disease	1163 (3.0)	551 (2.6)	1357 (4.6)	3071 (3.4)	< 0.001
Hypertension	18,381 (47.8)	9642 (45.3)	15,724 (53.7)	43,747 (49.1)	< 0.001
Parental history of diabetes	5899 (15.3)	3199 (15.0)	4865 (16.6)	13,963 (15.7)	< 0.001
Sleep duration, mean (SD), h/day	7.18 (0.94)	7.15 (0.93)	7.17 (1.06)	7.17 (0.98)	< 0.001
Sleep duration, h/day					
< 7	7821 (20.3)	4573 (21.5)	6977 (23.8)	19,371 (21.8)	< 0.001
7–8	28,440 (73.9)	15,671 (73.6)	20,072 (68.6)	64,183 (72.1)	
> 8	2220 (5.8)	1056 (5.0)	2214 (7.6)	5490 (6.2)	
Sedentary time, mean (SD), h/day	9.25 (1.70)	9.01 (1.77)	9.81 (1.85)	9.38 (1.80)	< 0.001
Sedentary time, h/day					
Low	13,503 (35.1)	8523 (40.0)	7421 (25.4)	29,447 (33.1)	< 0.001
Moderate	13,289 (34.5)	6995 (32.8)	9375 (32.0)	29,659 (33.3)	
High	11,689 (30.4)	5782 (27.1)	12,467 (42.6)	29,938 (33.6)	
MVPA, mean (SD), minutes/week	349.95 (186.94)	488.38 (269.08)	75.41 (44.68)	292.84 (243.25)	< 0.001

*£1 = €1.16, $1.24

### Association between PA pattern and incident T2D

**Fig. 3:**
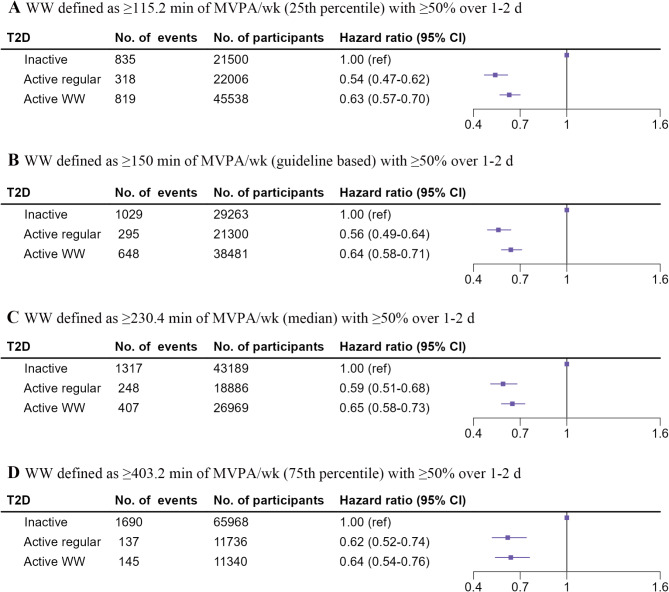
Relationships between PA patterns and the risk of developing T2D. **A** WW defined as ≥ 115.2 min of MVPA/wk (25th percentile) with ≥ 50% over 1–2 d, **B** WW defined as ≥ 150 min of MVPA/wk (guideline based) with ≥ 50% over 1–2 d, **C** WW defined as ≥ 230.4 min of MVPA/wk (median) with ≥ 50% over 1–2 d, **D** WW defined as ≥ 403.2 min of MVPA/wk (75th percentile) with ≥ 50% over 1–2 d. T2D = type 2 diabetes; WW = weekend warrior; MVPA = moderate-to-vigorous physical activity. The model was adjusted for age, sex, ethnicity, education status, household income, TDI, employment status, smoking status, drinking status, BMI, healthy dietary pattern status, sedentary time, sleep duration, cancer status, CVD status, hypertension status, and parental history of diabetes

**Fig. 4:**
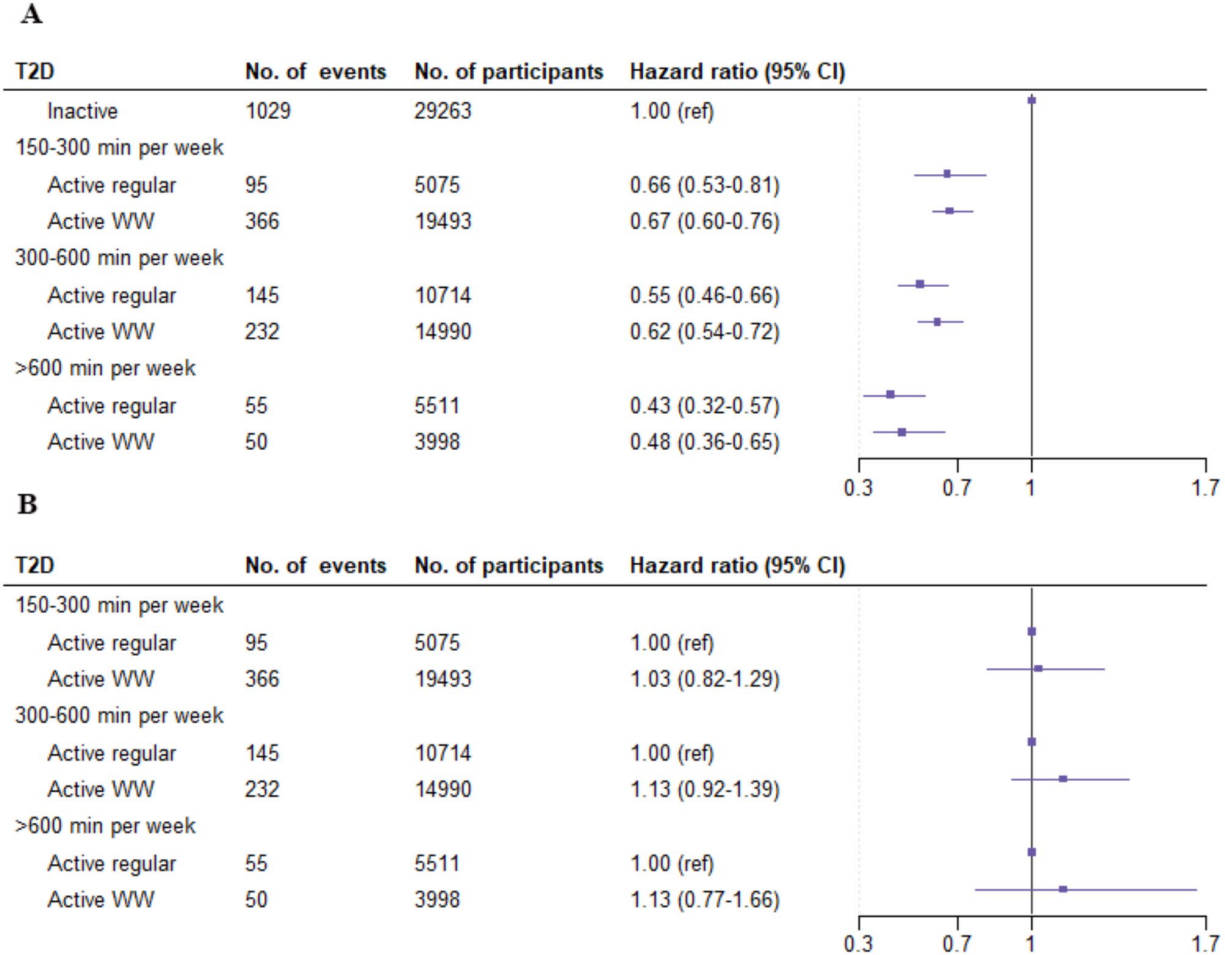
Relationships between PA patterns and the risk of developing T2D. **A** inactive regular group as the reference; **B** active regular group as the reference. T2D = type 2 diabetes; WW = weekend warrior; MVPA = moderate-to-vigorous physical activity. The model was adjusted for age, sex, ethnicity, education status, household income, TDI, employment status, smoking status, drinking status, BMI, healthy dietary pattern status, sedentary time, sleep duration, cancer status, CVD status, hypertension status, and parental history of diabetes

A total of 1972 participants developed T2D over a 7.9–year (interquartile range 7.4–8.4 years) median follow–up period. The relationships between PA patterns and T2D risk are shown in Fig. 3B. Active participants demonstrated a lower risk of T2D compared to inactive individuals. Specifically, in the fully adjusted models, the adjusted HRs (95% CIs) for T2D were 0.56 (0.49–0.64) for those engaging in regular activity and 0.64 (0.58–0.71) for the WWs, in comparison to the inactive group at the guideline-based threshold. Similarly, both the active groups consistently presented a significantly lower T2D risk compared to the inactive group across sample percentile thresholds. Figure 4 indicates that when the duration of MVPA was categorized into four levels, the risk of T2D progressively decreased as the duration increased. For the regular activity group, the adjusted HRs (95% CIs) for 150–300, 300–600, and ≥ 600 min/week were 0.66 (0.53–0.81), 0.55 (0.46–0.66), and 0.43 (0.32–0.57), respectively, compared to the inactive group. For the WW pattern, the adjusted HRs (95% CIs) for the same durations were 0.67 (0.60–0.76), 0.62 (0.54–0.72), and 0.48 (0.36–0.65), respectively. Furthermore, when examining the associations between the WW pattern and T2D risk stratified by MVPA duration per week, active WW and regular participants did not differ significantly in their risk of developing T2D across all MVPA strata.

### Stratified and sensitivity analyses

We conducted stratified analyses for associations of PA patterns with T2D incidence according to potential risk factors using the fully adjusted model. Our results showed that the associations between PA patterns with T2D incidence were not significantly modified by age, sex, obesity status, education status, current smoking status, current drinking status, CVD status, hypertension status, or sedentary time (all *P* > 0.0056) **(Table S3)**. The associations between PA patterns and incident T2D remained robust when individuals with T2D within the initial two years of follow-up were excluded **(Table S4)**, when individuals with missing covariates were excluded **(Table S5)**, and when the WW pattern was redefined based on MVPA concentration time distribution **(Table S6)**. Additionally, E values ranging from 2 to 4 in the study suggest that it is unlikely for a single unmeasured confounder to affect the stability of the results, as a correlation strength of 2–4 is relatively high. To be more specific, for example, the odds ratio for the association between active-regular vs. inactive and obesity was 1.40, and the HR for the association between obesity and incident diabetes was 3.08. These results indicate that the magnitude of overall unmeasured and residual confounding would need to be on the order of the observed effects of obesity to completely explain away the association between PA patterns and T2D risk **(Tables S4-S7 and S10)**.

## Discussion

In this large cohort study, we identified significant associations between adherence to both the WW and regular active patterns and a reduced risk of T2D, even among participants who did not meet the WHO recommended threshold of 150 min of MVPA per week. Our findings also indicate that exceeding current recommendations may confer even greater benefits. Furthermore, we performed subgroup analyses to examine several covariates related to T2D, revealing that these covariates did not significantly interact with PA patterns. This indicates that our findings are likely generalizable across diverse age groups and various health conditions such as cardiovascular diseases, hypertension, and smoking behaviors, transcending the specific characteristics initially considered.

Several studies examining the relationship between different PA patterns and health outcomes have indicated that WW patterns confer comparable benefits to regularly active pattern. For instance, three studies demonstrated that WWs achieved similar mortality reductions compared to those who distributed their exercise evenly throughout the week [18–20]. Additionally, observed cross-sectional relationships suggested that both the regularly active and WW groups may contribute to the prevention of metabolic syndrome [12]. However, the limitations of these studies stemmed from their design and reliance on self-reported PA. In contrast, accelerometry provides a more precise measurement of activity levels, mitigating recall bias and misclassification of PA intensity. Our study contributes to the existing literature by utilizing wrist-worn accelerometers to accurately capture the duration and frequency of MVPA from the UK Biobank accelerometer subcohort. To date, studies employing accelerometers to measure PA patterns have been limited, primarily focusing on associations between the WW pattern and outcomes such as brain health [14, 15, 17], mortality [13], and cardiovascular diseases [16]. Our findings illuminate the association between PA patterns, particularly the WW pattern, and the risk of developing T2D.

Utilizing accelerometer-measured PA data, we observed that 43.2% of participants engaged in a WW pattern, a significant increase compared to the 3.7% reported in previous studies based on self-report questionnaires [20]. Conversely, a population-based cohort study using accelerometry indicated that 32.3% of individuals were WWs, corroborating our findings [13]. This discrepancy suggests that device-measured data provide a more accurate assessment of PA patterns than self-reported data, consistent with prior research [11]. These results highlight the growing trend of the WW pattern among adults, likely attributable to hectic lifestyles and the convenience this pattern offers. Therefore, further research is warranted to explore the relationship between the WW pattern and various health outcomes, as well as to elucidate the potential health benefits associated with different PA patterns.

Our study indicates that both the WW and regularly active patterns confer similar benefits for T2D compared to inactive participants. These findings are consistent with previous studies demonstrating that active individuals possess a lower risk of T2D than their inactive counterparts [27, 36, 37]. A cohort study showed that high PA levels were negatively associated with diabetes risk [37]. Additionally, a meta-analysis suggested that any PA is preferable to none for T2D prevention, with increased activity further diminishing risk [36]. Importantly, our study emphasizes that WWs experience reduced T2D risk regardless of adherence to the recommended 150 min of MVPA weekly and illustrates that the risk for T2D decreases progressively with increased MVPA duration for both active patterns. This suggests that engaging in PA, even when concentrated into one to two days per week, may effectively mitigate T2D risk, with additional PA beyond the guidelines providing even greater reductions. Moreover, our subgroup analyses indicated no significant interactions between covariates and PA patterns, allowing us to conclude that the relationship between PA patterns and T2D remains consistent across diverse populations, indicating the applicability of our results to individuals with varying baseline characteristics.

The following mechanisms may elucidate the benefits of the WW pattern in relation to the risk of T2D. First, skeletal muscle is the primary tissue involved in insulin-stimulated glucose disposal and plays a crucial role in systemic glycemic control. Several studies have demonstrated that engaging in PA confers benefits for glycaemia [38] and insulin sensitivity [39], contributing to a protective effect against the development of T2D. Second, acute exercise sessions characteristic of the WW pattern have been associated with increased expression and secretion of interleukin-6, which helps combat chronic inflammation that can disrupt insulin signaling through insulin receptors [40, 41]. Future research could examine the mechanisms of the WW pattern versus more evenly distributed activity patterns on T2D risk, incorporating glucose and insulin biomarkers.

Our study has significant implications for T2D prevention. Both the regularly active and WW groups are similarly effective at preventing the onset of T2D, thereby allowing individuals to choose a pattern that aligns with their daily habits. However, there are drawbacks to the WW pattern. Evidence suggests that individuals who concentrate their weekly exercise into one or two days may face a higher risk of skeletal muscle injuries due to insufficient conditioning and lack of supplementary training [42]. Nonetheless, this risk appears comparable to that faced by individuals who follow a more evenly distributed exercise routine [42]. Consequently, further research is needed to better understand the potential negative effects associated with concentrated PA. Additionally, different intensities of MVPA may have varying effects on T2D risk. This study did not distinguish between the MVPA intensities of WWs and regularly active individuals. Future research exploring these intensity variations could reveal more nuanced health effects. Furthermore, future research could investigate whether a WW pattern exists among individuals with insufficient PA and explore whether such activity patterns offer health benefits comparable to those of regularly active individuals, thereby further enriching our understanding of how different PA patterns impact health.

### Strengths and limitations

Our study has several significant advantages. First, our study uniquely focused on the WW pattern and its association with T2D incidence, providing novel insights into this specific activity pattern and its potential impact on T2D risk through objective accelerometer-measured PA data. Notably, PA measured objectively through accelerometers mitigates misclassification and recall bias, which are often associated with self-reported data [43]. Second, we carefully considered multiple confounders and conducted several sensitivity analyses, including the calculation of E values [33], to quantify the potential impact of unmeasured confounders. This analysis provided insight into the robustness of our results, indicating that substantial unmeasured confounding would be needed to invalidate the observed associations.

However, this study is not without limitations. First, participants were invited to measure their PA over the course of a week, and it is possible that they altered their behavior during the observation period. Second, since the majority of participants in the UK Biobank study were white, with only a small proportion representing other racial groups, further research and long-term follow-up in more diverse populations and geographical regions are necessary to validate these findings and enhance generalizability. Third, PA information in this study was captured using a wrist-worn accelerometer, and additional research is required to ascertain whether PA data obtained via alternative methods are consistent with the findings of this study. Fourth, most covariates were self-reported and measured years prior to the introduction of accelerometers, which raises the possibility of recall bias and misclassification. Fifth, this study only controlled for sedentary behavior as a confounding factor. To specifically assess the impact of sedentary behavior, we suggest that future research explore the combined effects of MVPA and sedentary time to better understand the various behavioral factors that influence the incidence of T2D. Finally, although our analysis included a comprehensive range of confounders, the potential for residual or unmeasured confounding remains an inherent limitation of any observational study.

## Conclusions

Engaging in MVPA one or two days per week provides comparable benefits for the incidence of T2D as more evenly distributed activity. Moreover, exceeding current guidelines may offer even greater advantages. These findings are particularly relevant for individuals who face challenges in maintaining regular PA due to time constraints.

## Electronic supplementary material

Below is the link to the electronic supplementary material.

Supplementary Material 1

## Data Availability

The data analyzed in this study are available from the UK Biobank website with approved access. Data could be obtained upon direct application to the UK Biobank Study.

## Abbreviations

PA: Physical activity
T2D: Type 2 diabetes
WW: Weekend warrior
WWs: Weekend warriors
MVPA: Moderate-to-vigorous PA
HR: Hazard ratio
CI: Confidence interval
SD: Standard deviation
WHO: World Health Organization
UKB: UK Biobank
HES: Health Episode Statistics
SMR: Scottish Morbidity Records
ICD: International Classification of Diseases
TDI: Townsend Deprivation Index
BMI: Body mass index
CVD: Cardiovascular disease
