# Supplementary material for: Accelerometer-derived physical activity patterns and incident type 2 diabetes: a prospective cohort study
Source: Int J Behav Nutr Phys Act. 2025 Mar 31;22:38. doi: 10.1186/s12966-025-01734-7 (PMC11956271; doi:10.1186/s12966-025-01734-7)
Supplement: Supplementary file 1 — Supplementary Material 1 [file 12966_2025_1734_MOESM1_ESM.docx]

**Supplement material**

**Table S1** Covariate Definitions

**Table S2** Numbers (percentage) of the missing variables

**Table** **S3** Stratified analyses assessing the associations between PA pattern and type 2 diabetes

**Table S4** Associations of physical activity pattern with type 2 diabetes risk excluding cases in the initial two years of follow-up

**Table S5** Associations of physical activity pattern with type 2 diabetes risk in unimputed data

**Table S6** Associations of physical activity pattern with type 2 diabetes risk using activity threshold of≥150 minutes of MVPA per week (guideline-based)

**Table S7** The relationship between PA patterns and type 2 diabetes, stratified by the MVPA duration per week

**Table S8** The relationship between WW patterns and type 2 diabetes stratified by the MVPA duration per week, with active regular as the reference

**Table S9** The relationship between PA patterns and blood glucose level

**Table S10** Sensitivity analysis of physical activity patterns and type 2 diabetes risk by confounding factors.

**Table S1** Covariate Definitions

| Covariate | Field ID(s) | Field Name(s) | Coding | Note |
| --- | --- | --- | --- | --- |
| Age | 21022 | Age | Continuous age in years | age was calculated from the date of birth until the end date of accelerometer wear |
| Sex | 31 | Sex | 1. Male 2. Female |  |
| Body mass index | 21001 | Body mass index | Continuous in kg/m^2^ | BMI was equal to weight (kg) divided by the square of height (m^2^);  BMI <18.5: Underweight,  18.5≤BMI＜25: Normal weight,  25≤BMI＜30: Overweight,  BMI≥30: Obese. |
| Ethnicity | 21000 | Ethnic background | 1. White 2. Others |  |
| Education | 6138 | qualifications | 1. Degree 2. No degree |  |
| Townsend  Deprivation Index | 22189 | Townsend deprivation index at recruitment | Continuous scale | The Townsend Deprivation Index is a continuous composite area-level indicator of socioeconomic status and was assigned based on residential postcode, provided directly by the UK Biobank. |
| Household income | 738 | Average total household income before tax | 1. Less than 18,000 2. 18,000 to 30,999 3. 31,000 to 51,999 4. 52,000 to 100,000 5. Greater than 100,000 |  |
| Smoking status | 20116 | Smoking status | 1. Current 2. Previous 3. Never |  |
| Drinking status | 20117 | Alcohol drinker status | 1. Current 2. Previous 3. Never |  |
| Healthy  diet pattern | 1289, 1299, 1309, 1319, 1329, 1339, 1349, 1369, 1379,  1389 |  | 1. Yes 2. No | Healthy diet patterns were adapted from the American Heart Association Guidelines and defined as follows:  Total fruit and vegetable intake: > 4.5 pieces or servings a week. 3 Tablespoons of vegetables were considered one serving;  Total fish intake: > 2 per week;  Processed and red meat intake: 2 or fewer times intake of processed meat per week & 5 or fewer times intake of red meat per week;  The healthy diet score was dichotomised as 1 = at least two of the healthy food items, 0 = fewer than 2 of the healthy food items. |
| Employment status | 6142 | Current employment status | 1. Employed 2. Unemployed/Retired |  |
| Prevalent cancer | 2453 | Cancer diagnosed by doctor | 1. Yes 2. No |  |
| Prevalent cardiovascular disease | 6150 | Vascular/heart problems diagnosed by doctor | 1. Yes 2. No |  |
| Prevalent hypertension | 2966,  4080, 93,  4079, 94,  6153, 6177 |  | 1. Yes   2. No |  |
| Parental history of diabetes | 20107, 20110 | Illnesses of father,  Illnesses of mother | 1. Yes 2. No |  |
| Sleep duration | 1160 | Sleep duration |  |  |
| Sedentary time | 40047 | Sedentary - Overall average | Continuous age in h/day | accelerometer-measured, classified using a published machine-learning–based method.  divided into three groups according to the tertile:Low; Moderate; High |

**Table S2** Numbers (percentage) of the missing variables

| Variable | *n* | % |
| --- | --- | --- |
| Ethnicity | 306 | 0.34 |
| Education | 890 | 0.99 |
| TDI | 102 | 0.11 |
| household income | 9127 | 10.24 |
| Employment status | 206 | 0.23 |
| Smoking status | 232 | 0.26 |
| Drinking status | 83 | 0.09 |
| Healthy diet pattern | 14702 | 16.51 |
| Sleep duration | 258 | 0.29 |
| BMI | 186 | 0.21 |
| Prevalent cancer | 248 | 0.28 |

**Table** **S3** Stratified analyses assessing the associations between PA pattern and type 2 diabetes

| Characteristics | Participants | Inactive | Active WW |  | Active regular | *P* for interaction |
| --- | --- | --- | --- | --- | --- | --- |
|  |  |  | HR (95% CI) |  | HR (95% CI) |  |
| Sex |  |  |  |  |  | 0.878 |
| Female | 50912 | 1.00 (ref) | 0.66 (0.56-0.78) |  | 0.55 (0.44-0.69) |  |
| Male | 38132 | 1.00 (ref) | 0.64 (0.56-0.73) |  | 0.56 (0.47-0.66) |  |
| Age |  |  |  |  |  | 0.032 |
| ≥65 | 40153 | 1.00 (ref) | 0.67 (0.59-0.77) |  | 0.58 (0.49-0.70) |  |
| <65 | 48891 | 1.00 (ref) | 0.58 (0.49-0.69) |  | 0.50 (0.41-0.62) |  |
| Obesity |  |  |  |  |  | 0.277 |
| Yes | 16012 | 1.00 (ref) | 0.67 (0.57-0.78) |  | 0.56 (0.45-0.69) |  |
| No | 73032 | 1.00 (ref) | 0.60 (0.52-0.68) |  | 0.51 (0.43-0.61) |  |
| Education |  |  |  |  |  | 0.048 |
| Degree | 39061 | 1.00 (ref) | 0.57 (0.47-0.68) |  | 0.44 (0.35-0.56) |  |
| No Degree | 49983 | 1.00 (ref) | 0.68 (0.60-0.77) |  | 0.63 (0.53-0.74) |  |
| Current smoking |  |  |  |  |  | 0.749 |
| Yes | 6064 | 1.00 (ref) | 0.64 (0.45-0.90) |  | 0.64 (0.42-0.98) |  |
| No | 82980 | 1.00 (ref) | 0.64 (0.57-0.71) |  | 0.54 (0.47-0.62) |  |
| Current drinking |  |  |  |  |  | 0.428 |
| Yes | 84183 | 1.00 (ref) | 0.65 (0.58-0.72) |  | 0.54 (0.47-0.62) |  |
| No | 4861 | 1.00 (ref) | 0.61 (0.42-0.88) |  | 0.69 (0.46-1.03) |  |
| Cardiovascular disease |  |  |  |  |  | 0.649 |
| Yes | 3071 | 1.00 (ref) | 0.65 (0.47-0.90) |  | 0.47 (0.30-0.74) |  |
| No | 85973 | 1.00 (ref) | 0.64 (0.58-0.72) |  | 0.57 (0.49-0.65) |  |
| Hypertension |  |  |  |  |  | 0.154 |
| Yes | 43747 | 1.00 (ref) | 0.64 (0.57-0.73) |  | 0.59 (0.50-0.69) |  |
| No | 45297 | 1.00 (ref) | 0.63 (0.52-0.77) |  | 0.47 (0.36-0.61) |  |
| Sedentary time |  |  |  |  |  | 0.864 |
| High | 44459 | 1.00 (ref) | 0.62 (0.54-0.71) |  | 0.55 (0.46-0.65) |  |
| Low | 44585 | 1.00 (ref) | 0.67 (0.57-0.79) |  | 0.57 (0.47-0.71) |  |

HR=hazard ratio; WW=weekend warrior;

Model 1 was adjusted for age and sex;

Model 2 was further adjusted ethnicity, education status, household income, TDI, employment status, smoking status, drinking status, BMI, healthy dietary pattern status, sedentary time, sleep duration, cancer status, CVD status, hypertension status, and parental history of diabetes based on Model 1.

**Table S4** Associations of physical activity pattern with type 2 diabetes risk excluding cases in the initial two years of follow-up

| Physical  activity pattern | Model 1 | |  | Model 2 | | *E* value |
| --- | --- | --- | --- | --- | --- | --- |
|  | HR (95% CI) | *P* value |  | HR (95% CI) | *P* value |  |
| WW defined as ≥150 min of MVPA/wk (guideline based) with ≥50% over 1-2 d | | | | | | |
| Inactive | 1.00 (ref) | - |  | 1.00 (ref) | - |  |
| Active regular | 0.36 (0.31-0.41) | <0.001 |  | 0.54 (0.46-0.63) | <0.001 | 3.11 |
| Active WW | 0.44 (0.39-0.49) | <0.001 |  | 0.66 (0.58-0.74) | <0.001 | 2.40 |
| WW defined as ≥115.2 min of MVPA/wk (25th percentile) with ≥50% over 1-2 d | | | | | | |
| Inactive | 1.00 (ref) | - |  | 1.00 (ref) | - |  |
| Active regular | 0.34 (0.29-0.40) | <0.001 |  | 0.54 (0.46-0.63) | <0.001 | 3.11 |
| Active WW | 0.43 (0.39-0.48) | <0.001 |  | 0.66 (0.59-0.74) | <0.001 | 2.40 |
| WW defined as ≥230.4 min of MVPA/wk (median) with ≥50% over 1-2 d | | | | | | |
| Inactive | 1.00 (ref) | - |  | 1.00 (ref) | - |  |
| Active regular | 0.39 (0.33-0.45) | <0.001 |  | 0.57 (0.48-0.66) | <0.001 | 2.90 |
| Active WW | 0.44 (0.39-0.50) | <0.001 |  | 0.66 (0.58-0.75) | <0.001 | 2.40 |
| WW defined as ≥403.2 min of MVPA/wk (75th percentile) with ≥50% over 1-2 d | | | | | | |
| Inactive | 1.00 (ref) | - |  | 1.00 (ref) | - |  |
| Active regular | 0.43 (0.36-0.53) | <0.001 |  | 0.63 (0.51-0.76) | <0.001 | 2.55 |
| Active WW | 0.42 (0.34-0.51) | <0.001 |  | 0.62 (0.51-0.76) | <0.001 | 2.61 |

HR=hazard ratio; WW=weekend warrior;

Model 1 was adjusted for age and sex;

Model 2 was further adjusted ethnicity, education status, household income, TDI, employment status, smoking status, drinking status, BMI, healthy dietary pattern status, sedentary time, sleep duration, cancer status, CVD status, hypertension status, and parental history of diabetes based on Model 1.

**Table S5** Associations of physical activity pattern with type 2 diabetes risk in unimputed data

| Physical  activity pattern | Model 1 | |  | Model 2 | | *E* value |
| --- | --- | --- | --- | --- | --- | --- |
|  | HR (95% CI) | *P* value |  | HR (95% CI) | *P* value |  |
| WW defined as ≥150 min of MVPA/wk (guideline based) with ≥50% over 1-2 d | | | | | | |
| Inactive | 1.00 (ref) | - |  | 1.00 (ref) | - |  |
| Active regular | 0.34 (0.29-0.40) | <0.001 |  | 0.53 (0.45-0.62) | <0.001 | 3.18 |
| Active WW | 0.41 (0.36-0.46) | <0.001 |  | 0.62 (0.54-0.70) | <0.001 | 2.61 |
| WW defined as ≥115.2 min of MVPA/wk (25th percentile) with ≥50% over 1-2 d | | | | | | |
| Inactive | 1.00 (ref) | - |  | 1.00 (ref) | - |  |
| Active regular | 0.32 (0.27-0.38) | <0.001 |  | 0.52 (0.44-0.61) | <0.001 | 3.26 |
| Active WW | 0.40 (0.35-0.45) | <0.001 |  | 0.62 (0.55-0.70) | <0.001 | 2.61 |
| WW defined as ≥230.4 min of MVPA/wk (median) with ≥50% over 1-2 d | | | | | | |
| Inactive | 1.00 (ref) | - |  | 1.00 (ref) | - |  |
| Active regular | 0.39 (0.33-0.45) | <0.001 |  | 0.58 (0.49-0.69) | <0.001 | 2.84 |
| Active WW | 0.44 (0.38-0.50) | <0.001 |  | 0.66 (0.58-0.76) | <0.001 | 2.40 |
| WW defined as ≥403.2 min of MVPA/wk (75th percentile) with ≥50% over 1-2 d | | | | | | |
| Inactive | 1.00 (ref) | - |  | 1.00 (ref) | - |  |
| Active regular | 0.43 (0.35-0.53) | <0.001 |  | 0.64 (0.52-0.79) | <0.001 | 2.50 |
| Active WW | 0.42 (0.34-0.51) | <0.001 |  | 0.64 (0.52-0.78) | <0.001 | 2.50 |

HR=hazard ratio; WW=weekend warrior;

Model 1 was adjusted for age and sex;

Model 2 was further adjusted ethnicity, education status, household income, TDI, employment status, smoking status, drinking status, BMI, healthy dietary pattern status, sedentary time, sleep duration, cancer status, CVD status, hypertension status, and parental history of diabetes based on Model 1.

**Table S6** Associations of physical activity pattern with type 2 diabetes risk using activity threshold of≥150 minutes of MVPA per week (guideline-based)

| Physical  activity pattern | Model 1 | |  | Model 2 | | *E* value |
| --- | --- | --- | --- | --- | --- | --- |
|  | HR (95% CI) | *P* value |  | HR (95% CI) | *P* value |  |
| WW defined as ≥150 min of MVPA/wk (guideline based) with ≥50% over 1-2 d | | | | | | |
| Inactive | 1.00 (ref) | - |  | 1.00 (ref) | - |  |
| Active regular | 0.36 (0.32-0.41) | <0.001 |  | 0.56 (0.49-0.64) | <0.001 | 2.97 |
| Active WW | 0.42 (0.38-0.47) | <0.001 |  | 0.64 (0.58-0.71) | <0.001 | 2.50 |
| WW defined as ≥150 min of MVPA/wk (guideline based) with ≥75% over 1-2 d | | | | | | |
| Inactive | 1.00 (ref) | - |  | 1.00 (ref) | - |  |
| Active regular | 0.47 (0.39-0.56) | <0.001 |  | 0.60 (0.55-0.67) | <0.001 | 2.72 |
| Active WW | 0.47 (0.39-0.56) | <0.001 |  | 0.67 (0.56-0.81) | <0.001 | 2.35 |
| WW defined as ≥150 min of MVPA/wk (guideline based) with ≥50% over 1-2 consecutive days | | | | | | |
| Inactive | 1.00 (ref) | - |  | 1.00 (ref) | - |  |
| Active regular | 0.38 (0.34-0.43) | <0.001 |  | 0.59 (0.53-0.66) | <0.001 | 2.78 |
| Active WW | 0.43 (0.38-0.48) | <0.001 |  | 0.65 (0.57-0.73) | <0.001 | 2.45 |
| WW defined as ≥150 min of MVPA/wk (guideline based) with ≥50% MVPA over 1-2 weekend days | | | | | | |
| Inactive | 1.00 (ref) | - |  | 1.00 (ref) | - |  |
| Active regular | 0.41 (0.37-0.45) | <0.001 |  | 0.62 (0.57-0.69) | <0.001 | 2.61 |
| Active WW | 0.33 (0.27-0.41) | <0.001 |  | 0.53 (0.43-0.66) | <0.001 | 3.18 |

HR=hazard ratio; WW=weekend warrior;

Model 1 was adjusted for age and sex;

Model 2 was further adjusted ethnicity, education status, household income, TDI, employment status, smoking status, drinking status, BMI, healthy dietary pattern status, sedentary time, sleep duration, cancer status, CVD status, hypertension status, and parental history of diabetes based on Model 1.

**Table S7** The relationship between PA patterns and type 2 diabetes, stratified by the MVPA duration per week

| Physical  activity pattern | Model 1 | |  | Model 2 | | *E* value |
| --- | --- | --- | --- | --- | --- | --- |
|  | HR (95% CI) | *P* value |  | HR (95% CI) | *P* value |  |
| 150-300 min per week |  | | | | | |
| Inactive | 1.00 (ref) | - |  | 1.00 (ref) | - |  |
| Active regular | 0.52 (0.42-0.64) | <0.001 |  | 0.66 (0.53-0.81) | <0.001 | 2.40 |
| Active WW | 0.50 (0.44-0.56) | <0.001 |  | 0.67 (0.60-0.76) | <0.001 | 2.35 |
| 300-600 min per week |  | | | | |  |
| Inactive | 1.00 (ref) | - |  | 1.00 (ref) | - |  |
| Active regular | 0.36 (0.30-0.42) | <0.001 |  | 0.55 (0.46-0.66) | <0.001 | 3.04 |
| Active WW | 0.38 (0.33-0.43) | <0.001 |  | 0.62 (0.54-0.72) | <0.001 | 2.61 |
| >600 min per week |  | | | | |  |
| Inactive | 1.00 (ref) | - |  | 1.00 (ref) | - |  |
| Active regular | 0.24 (0.18-0.31) | <0.001 |  | 0.43 (0.32-0.57) | <0.001 | 4.08 |
| Active WW | 0.27 (0.20-0.36) | <0.001 |  | 0.48 (0.36-0.65) | <0.001 | 3.59 |

HR=hazard ratio; WW=weekend warrior;

Model 1 was adjusted for age and sex;

Model 2 was further adjusted ethnicity, education status, household income, TDI, employment status, smoking status, drinking status, BMI, healthy dietary pattern status, sedentary time, sleep duration, cancer status, CVD status, hypertension status, and parental history of diabetes based on Model 1.

**Table S8** The relationship between WW patterns and type 2 diabetes stratified by the MVPA duration per week, with active regular as the reference

| Physical  activity pattern | Model 1 | |  | Model 2 | |
| --- | --- | --- | --- | --- | --- |
|  | HR (95% CI) | *P* value |  | HR (95% CI) | *P* value |
| 150-300 min per week |  | | | | |
| Active regular | 1.00 (ref) | - |  | 1.00 (ref) | - |
| Active WW | 0.96 (0.76-1.20) | 0.693 |  | 1.03 (0.82-1.29) | 0.802 |
| 300-600 min per week |  | | | | |
| Active regular | 1.00 (ref) | - |  | 1.00 (ref) | - |
| Active WW | 1.06 (0.86-1.30) | 0.611 |  | 1.13 (0.92-1.39) | 0.241 |
| >600 min per week |  | | | | |
| Active regular | 1.00 (ref) | - |  | 1.00 (ref) | - |
| Active WW | 1.13 (0.77-1.66) | 0.531 |  | 1.13 (0.77-1.66) | 0.536 |

HR=hazard ratio; WW=weekend warrior;

Model 1 was adjusted for age and sex;

Model 2 was further adjusted ethnicity, education status, household income, TDI, employment status, smoking status, drinking status, BMI, healthy dietary pattern status, sedentary time, sleep duration, cancer status, CVD status, hypertension status, and parental history of diabetes based on Model 1.

**Table S9** The relationship between PA patterns and blood glucose level

| Physical activity pattern | OR (95% CI) | *P* value |
| --- | --- | --- |
| Inactive | 1.00 (ref) | - |
| Active regular | 0.97 (0.96-0.98) | <0.001 |
| Active WW | 0.97 (0.96-0.98) | <0.001 |

Model was adjusted for age and sex

HR=[odds ratio](https://zhida.zhihu.com/search?content_id=171948819&content_type=Article&match_order=1&q=odds+ratio&zhida_source=entity); WW=weekend warrior

**Table S10** Sensitivity analysis of physical activity patterns and type 2 diabetes risk by confounding factors.

| Smoking as confounding factor | | | | | | |
| --- | --- | --- | --- | --- | --- | --- |
|  | OR_EU_ | HR_UD_ | HR | B | HR_adjusted_ | E value |
| Inactive | 1.00 (ref) | | | | | |
| Active regular | 2.14 | 1.32 | 0.56 | 1.15 | 0.49 | 2.97 |
| Active WW | 1.92 | 1.32 | 0.64 | 1.13 | 0.57 | 2.50 |
| Obesity as confounding factor | | | | | | |
| Active regular | 1.40 | 3.08 | 0.56 | 1.24 | 0.45 | 2.97 |
| Active WW | 1.52 | 3.08 | 0.64 | 1.30 | 0.49 | 2.50 |
| Education as confounding factor | | | | | | |
| Active regular | 1.30 | 1.25 | 0.56 | 1.05 | 0.53 | 2.97 |
| Active WW | 1.23 | 1.25 | 0.64 | 1.04 | 0.62 | 2.50 |

OR_EU_:odd ratio. OR_EU_ denote the maximum odd ratio for any specific level of the confounders (obesity, smoking or education) comparing those with and without performing physical activity (Inactive vs. Active regular; or Inactive vs. Active WW), with adjustment already made for the measured covariates.

HR_UD_: Hazard ratio.HR_UD_ denote the maximum hazard ratio for the outcome comparing any 2 categories of the confounders (obesity, smoking or education), within either physical activity group, conditional on the observed covariates.

HR: the association between physical activity patterns and incident type 2 diabetes.

B: denote the largest factor by which the observed relative risk could be altered by unmeasured confounders of a particular strength. B=OR_EU_HR_UD_/(OR_EU_+HR_UD_-1).

HR_adjusted_=HR/B.

E value: the minimum strength of association, on the risk ratio scale, that an unmeasured confounder would need to have with both the treatment and outcome, conditional on the measured covariates, to explain away a treatment–outcome association.
